# A rapid and efficient in vitro regeneration system for lettuce (*Lactuca sativa* L.)

**DOI:** 10.1186/s13007-017-0208-0

**Published:** 2017-07-21

**Authors:** Isabel Armas, Natalia Pogrebnyak, Ilya Raskin

**Affiliations:** Department of Plant Biology, School of Environmental and Biological Sciences, Rutgers, The State University of New Jersey, New Brunswick, NJ 08901 USA

**Keywords:** *Lactuca sativa*, Tissue culture, Shoot induction, Regeneration efficiency, Activated charcoal

## Abstract

**Background:**

Successful biotechnological improvement of crop plants requires a reliable and efficient in vitro regeneration system. Lettuce (*Lactuca sativa* L.), one the most important vegetable crops worldwide, is strongly genotype-dependent in terms of regeneration capacity, limiting the potential for biotechnological improvement of cultivars which show recalcitrance under currently available protocols. The effect of different nutrient sources, plant hormone combinations and activated charcoal supplementation on shoot induction efficiency was evaluated on the cultivar ‘RSL NFR’, which had previously shown poor regeneration efficiency.

**Results:**

Multiple shoot organogenesis from cotyledon explants was recorded at the highest frequency and speed on Murashige and Skoog regeneration medium supplemented with 200 mg/l of activated charcoal, 3% sucrose, 10 mg/l benzylaminopurine and 0.5 mg/l naphthaleneacetic acid, which induced shoots through direct regeneration in 90.8 ± 7.9% of explants. High shoot induction efficiency was also observed, albeit not quantified, when using this medium on some other cultivars.

**Conclusions:**

This activated charcoal-containing regeneration medium might offer a rapid and efficient option for direct shoot induction in some lettuce genotypes that do not respond well to common lettuce regeneration protocols. This is also the first report of the effect of activated charcoal in lettuce tissue culture.

**Electronic supplementary material:**

The online version of this article (doi:10.1186/s13007-017-0208-0) contains supplementary material, which is available to authorized users.

## Background

Lettuce (*Lactuca sativa* L.) is a leafy herbaceous self-pollinated annual plant of the family Asteraceae, grown worldwide for consumption, most commonly raw, as a salad green. The Food and Agriculture Organization of the United Nations estimated worldwide production of lettuce (combined with chicory) to be almost 25 million metric tons in 2014, with a gross production value close to US$ 12,000 million as of 2012 [1]. Lettuce is therefore a good candidate for agronomic trait improvement, including the delivery of nutrients and bioactive compounds beneficial to human health. Following this line of reasoning, lettuce varieties with traits such as herbicide resistance [2], virus resistance [3], environmental stress resistance [4, 5], yield enhancement [6], accumulation of pharmaceutical proteins [7, 8], increased leaf calcium content [9], and increased total polyphenol content [10, 11], have been developed.

Successful plant genetic engineering, as well as of some non-transgenic approaches such as screening plantlets for a desired phenotype using an in vitro platform, ultimately rest on the ability to regenerate whole plants reliably and efficiently through tissue culture. A regeneration system which induces rapid multiple shoot production, without a slow intermediate callus stage, is preferred for most applications. The progress and efficiency of regeneration can be influenced by three major factors: selection of a cultivar with adequate regeneration efficiency, optimization of explant source, and adaptation of the medium for the given genotype [12–14]. Medium optimization involves selecting a salt and vitamin mix, a carbon source, and the most adequate hormone combination. It may also be possible to further optimize media by adding supplements which induce beneficial effects on regeneration even if they are not strictly necessary, due to their additional nutrient content or their physicochemical properties, such as the addition of coconut water, potato extract, banana homogenate or algal compounds to orchid tissue culture media [15].

In the case of lettuce the regeneration response has been reported to be highly variable across experiments and dependent on genotype [12, 13, 16], with many cultivars showing strong recalcitrance. Shoot organogenesis can be induced by culturing 3–7 day old cotyledon explants on a variety of solid media, and different hormone combinations have been evaluated over the years for the best regeneration results [12–14, 17–23]. One of the most common approaches uses a medium defined by Hunter and Burritt [21] which is reported to produce up to 72% direct shoot regeneration [14], containing 0.09 mg/l (0.44 µM) of the cytokinin benzylaminopurine (BA) and 0.1 mg/l (0.54 µM) of the auxin naphthaleneacetic acid (NAA), or minor variations [22, 23]. Nevertheless none of the available regeneration protocols offer consistent results across all cultivars, making optimization on a case-by-case basis critical for the improvement of recalcitrant genotypes.

Improvement of cultivar ‘RSL NFR’ through phenotype selection in vitro was delayed by the low shoot induction rates obtained using an in-house medium containing Murashige and Skoog (MS) salt mixture with Gamborg vitamins, 3% sucrose, 2 mg/l BA and 0.1 mg/l NAA. Regeneration took place through an intermediate callus stage, with shoots developing 4–6 weeks after cotyledon excision and no more than 10% of explants producing shoots (unpublished data). In the present study we attempted to optimize medium composition to induce fast multiple shoot induction, and also healthy shoots, in ‘RSL NFR’. The relatively low efficiency medium originally used was tested side by side with each variation as a control. All variations were tested on ‘RSL NFR’ and on ‘Winter Density’, a cultivar where high callus production and very low shoot induction efficiency had been observed. A strong and consistent improvement in multiple shoot induction frequency and speed was observed on regeneration medium supplemented with 200 mg/l activated charcoal (AC), observable as early as 10 days after explant excision.

## Methods

### Chemicals and reagents

Murashige and Skoog modified basal salt mixture with Gamborg vitamins, Schenk and Hildebrandt modified basal salt mixture with vitamins, micropropagation grade agar, benzylaminopurine solution, kinetin solution, thidiazuron solution, zeatin solution, naphthaleneacetic acid solution and indolebutyric acid solution were purchased from PhytoTechnology Laboratories (Overland Park, KS, USA). Activated charcoal, glucose and sucrose were purchased from Sigma (St. Louis, MO, USA).

### Tissue culture conditions

Seeds of loose leaf ‘RSL NFR’ lettuce provided by Shamrock Seed Company (Salinas, CA, USA) and of romaine ‘Winter Density’ lettuce (Johnny’s Selected Seeds, Winslow, ME, USA) were surface sterilized by immersion in 70% ethanol for 1 min, followed by a 1.2% sodium hypochlorite solution for 12 min and rinsed three times with sterilized distilled water. Surface sterilized seeds were placed into deep Petri dishes (VWR, Radnor, PA, USA) containing 40 ml of germination medium solidified with 7 g/l agar (Table 1). Three days after germination the cotyledons were aseptically excised and placed into deep Petri dishes containing 40 ml of different types of shoot induction media solidified with 7 g/l agar (Tables 2, 3). Petri dishes were kept inside a GC-96 CW walk-in environmentally controlled growth chamber (EGC, Chagrin Falls, OH, USA) at 22 °C, under a photosynthetically active radiation (PAR) light intensity of 10.6 ± 1.7 mol/m^2^ day provided by full spectrum 32W Philips F32T8/DX fluorescent lamps (Philips, Andover, MA, USA) and a 16/8 h (light/dark) photoperiod. Petri dishes were checked weekly for any changes.

**Table 1:** Tissue culture media originally used on ‘RSL NFR’

Medium	Basal salt mix	Sugar	Cytokinins (mg/l)	Auxins (mg/l)
Germination	MS 1/2×	Sucrose 1%	–	–
Shoot induction	MS 1×	Sucrose 3%	BA 2	NAA 0.1
Root induction	MS 1×	Sucrose 3%	–	IBA 1

All media pH was adjusted to 5.7, solidified with 7 g/l agar and autoclaved at 121 °C and 103 kPa for 20 min. Shoot induction medium was used as a control for all optimization experiments

*MS* Murashige and Skoog modified basal salts with Gamborg vitamins, *BA* 6-benzylaminopurine, *NAA* naphthaleneacetic acid, *IBA* indole-3-butyric acid

**Table 2:** Nutritional and hormonal variations of regeneration media

Medium	Basal salt mix	Sugar	Cytokinins (mg/l)	Auxins (mg/l)
Control	MS 1×	Sucrose 3%	BA 2	NAA 0.1
M1	SH 1×	Sucrose 3%	BA 2	NAA 0.1
M2	MS 1×	Glucose 1.5%	BA 2	NAA 0.1
M3	MS 1×	Sucrose 3%	BA 0.09	NAA 0.1
M4	MS 1×	Sucrose 3%	KIN 2	NAA 0.1
M5	MS 1×	Sucrose 3%	TDZ 2	NAA 0.1
M6	MS 1×	Sucrose 3%	ZEA 2	NAA 0.1
M7	MS 1×	Sucrose 3%	BA 1	NAA 0.05
M8	MS 1×	Sucrose 3%	BA 3	NAA 0.15
M9	MS 1×	Sucrose 3%	BA 4	NAA 0.2
M10	MS 1×	Sucrose 3%	BA 5	NAA 0.25
M11	MS 1×	Sucrose 3%	BA 6	NAA 0.3
M12	MS 1×	Sucrose 3%	BA 7	NAA 0.35
M13	MS 1×	Sucrose 3%	BA 8	NAA 0.4
M14	MS 1×	Sucrose 3%	BA 9	NAA 0.45

All media pH was adjusted to 5.7, solidified with 7 g/l agar and autoclaved at 121 °C and 103 kPa for 20 min

*MS* Murashige and Skoog modified basal salts with Gamborg vitamins, *SH* Schenk and Hildebrandt modified basal salts with vitamins, *BA* benzylaminopurine, *KIN* kinetin, *TDZ* thidiazuron, *ZEA* zeatin, *NAA* naphthaleneacetic acid

**Table 3:** Activated charcoal supplementation of regeneration media

Medium	Basal salt mix	Sugar	Cytokinins (mg/l)	Auxins (mg/l)	AC (mg/l)
Control	MS 1×	Sucrose 3%	BA 2	NAA 0.1	–
M15	MS 1×	Sucrose 3%	BA 2.5	NAA 0.125	20
M16	MS 1×	Sucrose 3%	BA 5	NAA 0.25	20
M17	MS 1×	Sucrose 3%	BA 2.5	NAA 0.125	200
M18	MS 1×	Sucrose 3%	BA 5	NAA 0.25	200
M19	MS 1×	Sucrose 3%	BA 10	NAA 0.5	200
M20	MS 1×	Sucrose 3%	BA 5	NAA 0.25	2000
M21	MS 1×	Sucrose 3%	BA 10	NAA 0.5	2000

All media pH was adjusted to 5.7, solidified with 7 g/l agar and autoclaved at 121 °C and 103 kPa for 20 min

*AC* activated charcoal, *MS* Murashige and Skoog modified basal salts with Gamborg vitamins, *BA* benzylaminopurine, *NAA* naphthaleneacetic acid

### Experimental design and analysis of results

Each treatment group comprised 15 cotyledon explants contained in a single Petri dish and all treatments were replicated a minimum of three times, totaling at least N = 45 explants per treatment group. The earliest signs of shoot induction were recorded, and the effects of each medium on shoot induction rate and shoot quality were evaluated at weeks 6 and 8. The percentage of explants with at least one shoot was recorded for each treatment group, and the size, growth speed, morphology and health conditions of shoots were evaluated visually. The means and standard deviations (SD) were calculated from pooled data and all results are expressed as mean ± SD. The statistical significance of the differential effects of treatments was determined using a general linear model analysis of variance (ANOVA) followed by a Dunnett test. A global significance level was set at α = 0.05. All statistical procedures were performed using GraphPad Prism 6 (GraphPad Software Inc., La Jolla, CA, USA).

## Results

### Nutritional and hormonal variations of the shoot induction medium

**Fig. 1:**
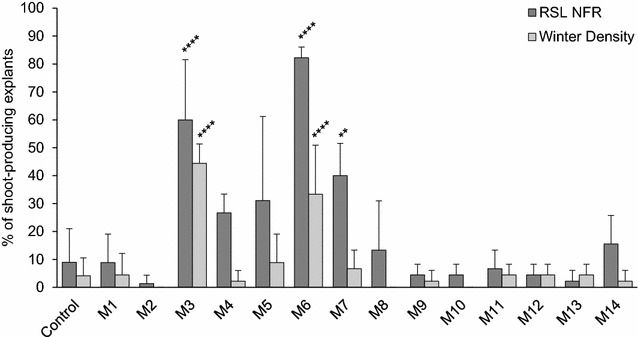
Shoot induction in cultivars ‘RSL NFR’ and ‘Winter Density’ under different nutritional and hormonal conditions but no AC. The number of explants which produced at least one shoot 8 weeks after excision were quantified for all treatment groups and expressed as a percentage of the total explant number. A minimum of N = 45 explants were evaluated per treatment group. Data were pooled and are presented as mean ± SD. Significant differences between the control group and each treatment group were analyzed by a one-way ANOVA followed by a Dunnett test (**p ≤ 0.01, ****p ≤ 0.0001). Data does not reflect size, growth speed or overall quality of the shoots

**Fig. 2:**
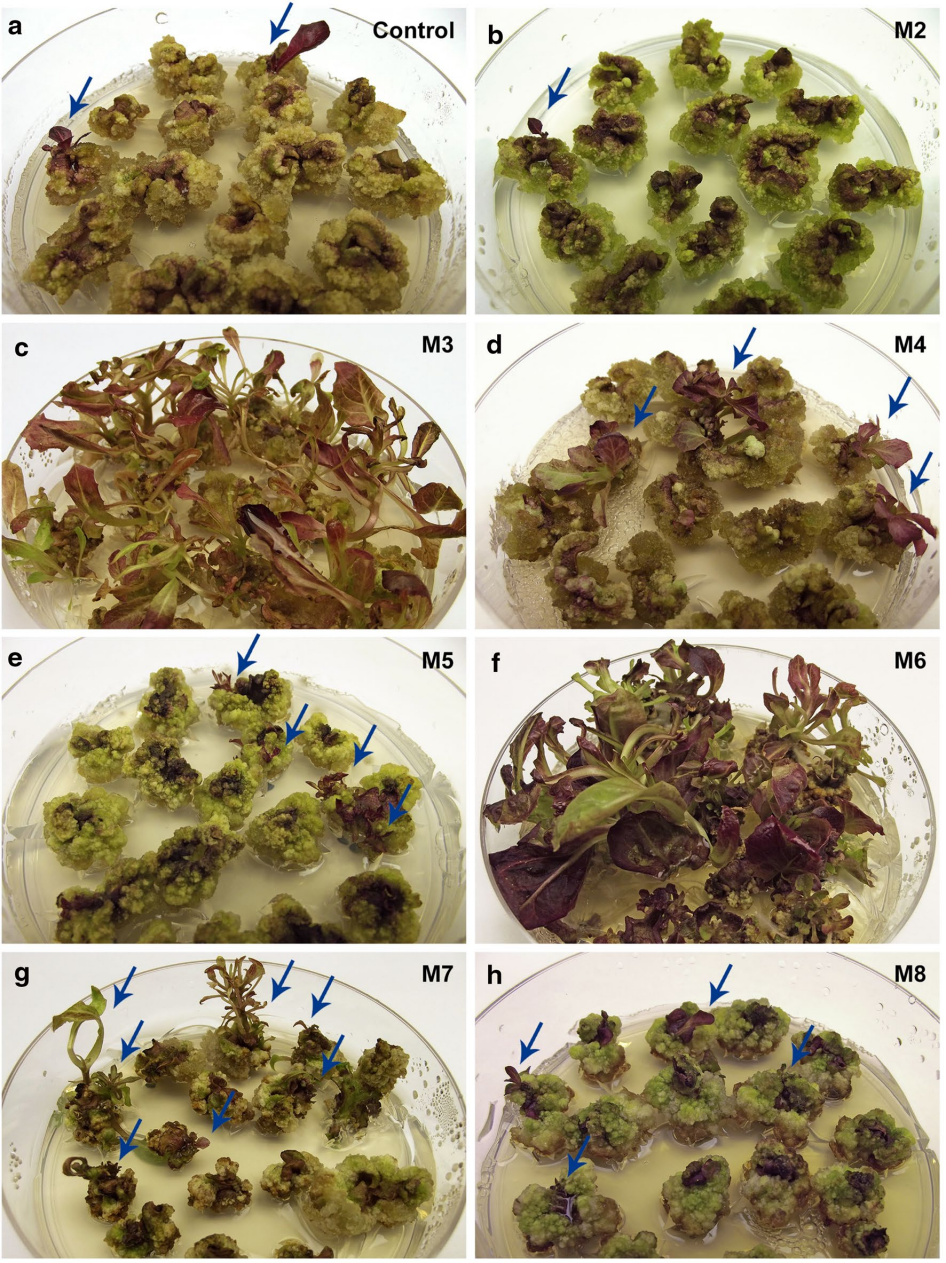
Visual comparison of shoots induced under different nutritional and hormonal conditions but no AC. No significant differences in shoot count, size and growth speed were observed between the control medium (**a**) and media M2 (**b**), M4 (**d**), M5 (**e**), or M8–M14 (**h**). Significant improvements were observed on media M3 (**c**) and M6 (**f**), and to a lesser degree also in M7 (**g**); however in all cases the shoots appeared weak, etiolated, or showed an aberrant morphology when compared to the control. All images are of cultivar ‘RSL NFR’ at week 7. Shoots which may be hard to discern are *marked with arrows*

Modification of the salt mix and sugar components of the original medium yielded no significant changes when compared to the control medium (Fig. 1, M1–M2). Substituting the Murashige and Skoog (MS) basal salt mixture for Schenk and Hildebrandt (SH) basal salt mixture (Table 2, M1), sometimes used in lettuce shoot induction [12, 13, 20], produced no changes. A 1992 study by Teng et al. concluded that a regeneration medium containing glucose induces more shoot induction and less callus growth in lettuce than a regeneration medium containing sucrose [20]. However our results show no differences (Table 2, M2) aside from the development of hyperhydric calli (Fig. 2b), which has been previously correlated with an overall decrease in regeneration efficiency.

On the other hand, changes to the hormonal composition of the original medium affected regeneration efficiency (Fig. 1, M3–M14), especially in cultivar ‘RSL NFR’, where shoot induction was overall higher and more responsive to medium modifications than in cultivar ‘Winter Density’. The medium defined by Hunter and Burritt [21] (Table 2, M3) induced a significant increase in shoot count, size and growth speed, with 60 ± 21.5% of ‘RSL NFR’ explants producing at least one shoot. These shoots developed directly from explant tissue 2 weeks after excision, in marked contrast to the control. However, the morphology observed for these shoots was markedly abnormal with typical symptoms of etiolation: elongated stems with very thin and narrow leaf blades, chlorosis and overall weakness (Fig. 2c).

Substitution of the cytokinin BA for kinetin or thidiazuron (TDZ) (Table 2, M4–M5) induced minor changes which were not statistically significant. Shoots induced by kinetin were comparable in size, growth speed and morphology (Fig. 2d) to those in the control group, whereas shoots induced by TDZ were smaller and weaker (Fig. 2e); regeneration was in all cases slow and mediated through a callus stage. Zeatin (Table 2, M6), however, induced a significant increase in shoot count, size and growth speed, with 82.2 ± 3.8% of ‘RSL NFR’ explants producing at least one shoot. These shoots developed directly from explant tissue 2 weeks after excision, being later followed by a moderate amount of roots and callus. These shoots, however, showed aberrant morphology, with elongated stems and very irregularly shaped leaf blades (Fig. 2f).

A range of hormone concentrations, from 1 mg/l BA and 0.05 mg/l NAA to 9 mg/l BA and 0.45 mg/l NAA, were also evaluated (Table 2, M7–M14). A significant increase in shoot induction was observed for ‘RSL NFR’ grown on medium M7. These shoots developed directly from explant tissue 2 weeks after excision, but overall looked weak, growing very slowly and showing symptoms of etiolation (Fig. 2g). Regeneration on media M8–M14 was very low, with all shoots developing indirectly from an intermediate callus stage, growing very slowly and often arresting their growth altogether (Fig. 2h).

### Supplementation of the shoot induction medium with activated charcoal

The range of AC concentrations used in plant tissue culture is highly variable, from reports of Brassica microspore embryogenesis being supplemented with 150 g/l AC [24], to 0.002 g/l AC [25, 26] being used for root induction in *Capsicum* sp. and *Aesculus* sp. Additionally, it has been shown that a large proportion of the hormones present in the regeneration medium are very quickly adsorbed by the AC particles, making it difficult to know the amounts and ratios which are available to the plant tissue [27–31]. Medium supplementation with AC therefore requires careful optimization of the relative amounts of both AC and hormones.

**Fig. 3:**
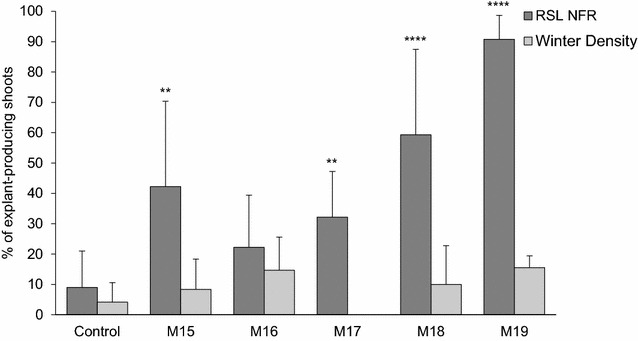
Shoot induction in cultivars ‘RSL NFR’ and ‘Winter Density’ under increasing concentrations of AC. The number of explants which produced at least one shoot 8 weeks after excision were quantified for all treatment groups and expressed as a percentage of the total explant number. A minimum of N = 45 explants were evaluated per treatment group, and a minimum of N = 120 explants were evaluated for treatment groups M17, M18 and M19. Data were pooled and are presented as mean ± SD. Significant differences between the control group and each treatment group were analyzed by a one-way ANOVA followed by a Dunnett test (**p ≤ 0.01, ****p ≤ 0.0001). Data does not reflect size, growth speed or overall quality of the shoots. Media M20 and M21 not shown since no shoots were produced for either cultivar. Media M17 not shown for cultivar ‘WD’ since it was tested only on cultivar ‘RSL NFR’

**Fig. 4:**
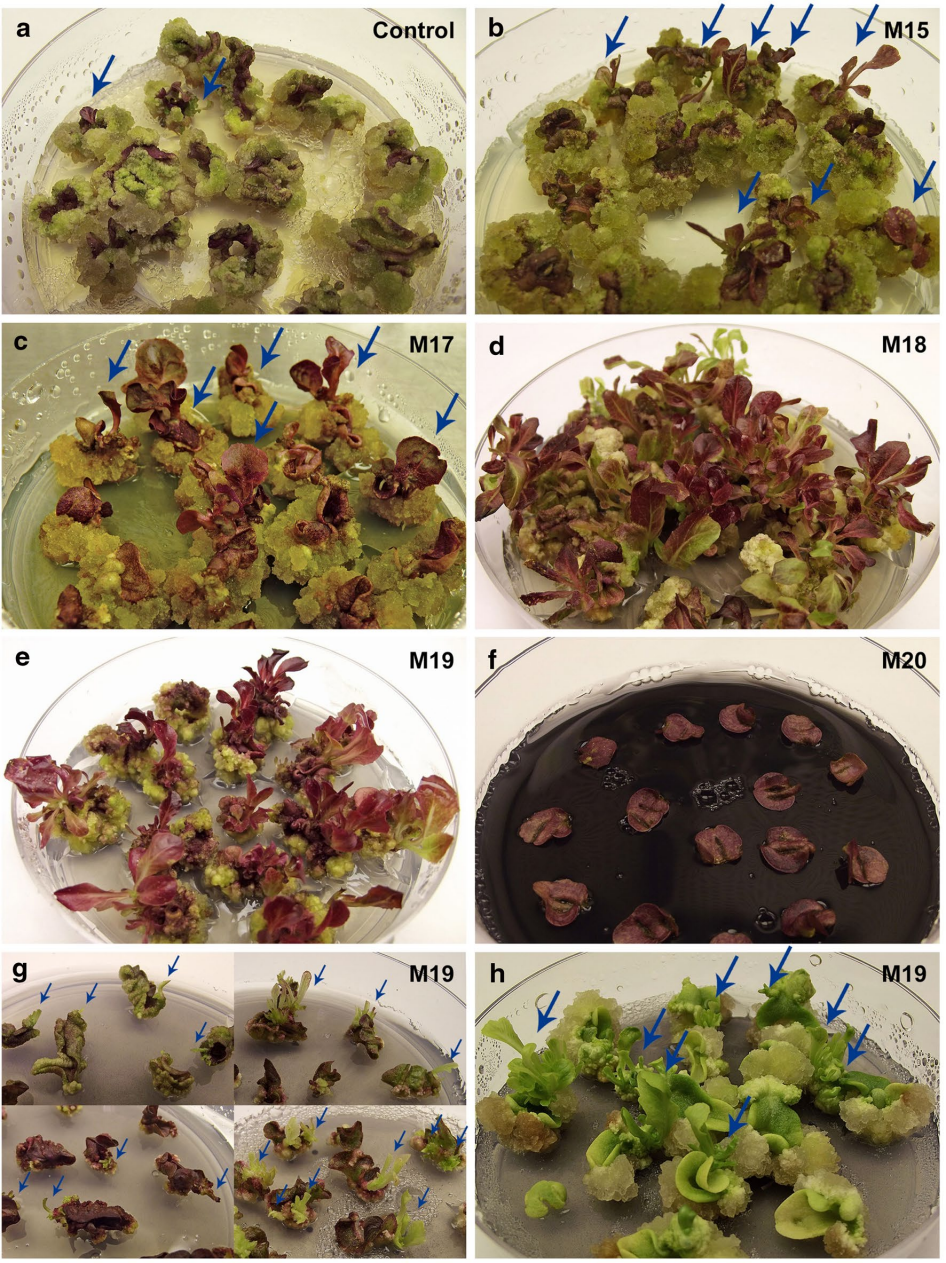
Visual comparison of shoots induced under increasing concentrations of AC. A significant increase in shoot count, size and growth speed over the control medium (**a**) was observed on media M15 (**b**), M17 (**c**), and especially M18 (**d**) and M19 (**e**). Tissue did not react on media M20 and M21 (**f**). All observed shoots were produced through direct regeneration, initiating development from explant tissue between days 10 and 14 (**g**), and grew at a moderate speed with normal morphology. All images are of cultivar ‘RSL NFR’, except for **h** which shows cultivar ‘Bambi’. All treatments are shown at week 7, except for **g** which shows direct initiation of shoot development at week 2. Shoots which may be hard to discern are *marked with arrows*

AC was incorporated to the medium in a concentration of 20, 200 and 2000 mg/l, while increasing the amounts of BA and NAA proportionally to compensate for their adsorption. Significant differences were observed between treatments in cultivar ‘RSL NFR’ (Fig. 3). The combination of 20 mg/l AC with 2.5 mg/l BA and 0.125 mg/l NAA (Table 3, M15) produced a significant increase in shoot count, size and growth speed when compared to the control medium with no AC (Fig. 4b). However, the same amount of charcoal combined with 5 mg/l BA and 0.25 mg/l NAA (Table 3, M16) induced no significant changes. Addition of 200 mg/l AC produced a strong and significant increase in shoot count, size and development speed on media containing either 5 mg/l BA and 0.25 mg/l NAA (Table 3, M18) or 10 mg/l BA and 0.5 mg/l NAA (Table 3, M19). These shoots initiated development directly from explant tissue around day 10, and almost all explants produced multiple shoots (Fig. 4d, e). Addition of 2000 mg/l AC (Table 3, M20–M21), however, induced no reaction in the tissue regardless of the hormonal dose (Fig. 4f), most likely due to a lack of available hormones due to their complete adsorption by the AC particles present. Media 15, 16, 18, 19, 20 and 21 were evaluated again in a second set of identical experiments which produced very similar results. Overall, rapid and multiple shoot induction was recorded from 64 ± 37% of all ‘RSL NFR’ explants in medium M18 and 86.7 ± 9.4% of all ‘RSL NFR’ explants in medium M19.

We attempted to further adjust the hormonal dose added to media containing 200 mg/l AC by comparing media M18 and M19 to a medium containing 2.5 mg/l BA and 0.125 mg/l NAA (Table 3, M17). The results confirmed the trend observed in the previous experiments and showed a clear dose–response effect, with increased hormone content leading to increased shoot induction efficiency within the tested range (Fig. 3). Shoots initiated development directly from explant tissue between day 10 and 14 (Fig. 4g), often appeared in bundles of multiple shoots and showed normal morphology, although variability between Petri dishes in terms of shoot size and growth speed was higher than in previous experiments. Medium M17 induced healthy shoot formation from 32.2 ± 15% of ‘RSL NFR’ explants (Fig. 4c), a significant increase from the 9 ± 12% recorded under control conditions, yet much lower than the cumulative final values for media M18 and M19, 59.3 ± 28.2 and 90.8 ± 7.9% respectively. Comparison of results across all Petri dishes and experiment repeats showed the highest result consistency for medium M19 (see shoot induction rates per individual Petri dish in Additional file 1).

Despite AC supplementation not inducing any statistically significant changes in the percentage of explants which produced shoots in cultivar ‘Winter Density’ (Fig. 3), it must be noted that most regenerated shoots started direct development around week 2, in strong contrast to the control where shoots developed through an intermediate callus stage. High frequency direct regeneration on medium M19 was also observed, even if not quantified, in cultivars ‘Paris White’, ‘Lettony’, ‘Simpson Elite’ and ‘Bambi’ (Fig. 4h).

## Discussion

It has been long known that in vitro regeneration of lettuce can take months if using an inadequate combination of explant source and medium composition [20], limiting its potential for biotechnological improvement. Given how regeneration efficiency in lettuce is statistically dependent on genotype [12, 13, 16] it may not be possible to define a single medium that induces high levels of shoot induction across all existing cultivars. Different studies in lettuce tissue culture have focused on ranking different cultivars according to their callus formation or direct shoot induction capacities [12, 22]. Situations where the cultivar of interest must be chosen according to different criteria, however, make a strong case for the development of alternative regeneration conditions for recalcitrant cultivars.

In ‘RSL NFR’, medium M19 consistently increased shoot organogenesis from approximately 10% of explants producing single shoots in the control medium, to multiple shoot induction from at least 70% of explants in all individual Petri dishes evaluated, and up to an average of 90%. This medium also decreased the time required for initiation of shoot organogenesis from up to 6 weeks in the control medium to under 2 weeks. These shoots developed roots successfully when transferred to root induction medium, the plantlets survived transplantation to potting mix, and the adult plants were able to produce viable seeds. Observation of the effects of medium M19 on other loose leaf and romaine lettuce cultivars showed that the beneficial effect of AC is still conditioned by the lettuce genotype, with some of the genotypes barely modifying their previous response when under the presence of AC, and with others showing direct and rapid shoot induction, high frequency of shoot organogenesis, or both. Despite the lack of a uniform response, we believe our results highlight the worth of testing M19 or in general any AC-containing media when attempting regeneration of recalcitrant cultivars. It may be of value, for example, to test a range of AC concentrations on the commonly used Hunter and Burritt medium (M3), or on media containing zeatin (M6).

Given the lack of significant changes observed in media containing increased amounts of total BA and NAA (M8–M14) it seems unlikely that the results observed in media M18 and M19 are solely influenced by the high hormone levels. A more likely reason behind the observed improvement would be the physicochemical properties of AC, which is processed to have a large network of very fine pores, maximizing its surface area and adsorption capacity. AC is a common supplement in tissue culture for many plant species as it has been observed to improve cell growth and development and enhance micropropagation, somatic embryogenesis, protoplast culture, anther and microspore culture, seed germination and root formation [31, 32]. The mechanism of this beneficial effect is still unclear, but it has been hypothesized to be a combination of adsorbing inhibitory substances released during tissue culture [27, 33–35], decreasing accumulation of phenolic exudates [36, 37], improving medium pH [38], providing a dark environment which mimics soil conditions [39], and progressively releasing beneficial substances naturally present in the AC particles [40, 41].

Some improvements in shoot regeneration efficiency of ‘RSL NFR’ were also observed on media M3, M6 and M7, which are consistent with rates of up to 72% previously reported for other cultivars on medium M3 [14], with reports of zeatin being the most potent cytokinin, and with some reports that low amounts of BA induce a decrease in callus production and an increase in direct shoot induction [14, 22], respectively. Nevertheless, these studies make little mention to the overall quality of the shoots induced and whether an etiolated or abnormal morphology is present. It must be taken into account that shoot weakness can decrease plantlet survival and subsequent seed production.

## Conclusions

Overall and out of all combinations tested, AC-enriched medium M19 produced the highest number of strong and healthy shoots as well as the most consistent results on recalcitrant cultivar ‘RSL NFR’. M19 or small variations thereof might therefore pose a valuable alternative for fast and efficient multiple shoot production in lettuce cultivars which do not respond well to common media, potentially opening new opportunities for the biotechnological improvement of lettuce. This is also the first report of the use and beneficial effects of activated charcoal in lettuce tissue culture.

## Additional file

**Additional file 1.** A spreadsheet containing all raw data generated and analyzed during this study. The first sheet contains the data for cultivar ‘RSL NFR’ and the second sheet contains the data for cultivar ‘Winter Density’.

## Abbreviations

AC: activated charcoal
BA: benzylaminopurine
IBA: indolebutyric acid
MS: Murashige and Skoog basal salt mixture
NAA: naphthaleneacetic acid
PAR: photosynthetically active radiation
SD: standard deviation
SH: Schenk and Hildebrandt basal salt mixture
TDZ: thidiazuron
